# The necessary and sufficient mechanism of consciousness in a layered mind

**DOI:** 10.3389/fpsyg.2023.1280959

**Published:** 2023-09-28

**Authors:** Zenan Ruan

**Affiliations:** ^1^Center for the Study of Language and Cognition, School of Philosophy, Zhejiang University, Hangzhou, China; ^2^Department of Automation, School of Mechanical Engineering and Automation, Zhejiang SCI-TECH University, Hangzhou, China

**Keywords:** consciousness, Gazzaniga, theories of consciousness, IIT, GNWT

## Introduction

The study of consciousness is becoming one of several significant challenges at the frontiers of science, in contrast to its previously being off-limits. With the application of binocular rivalry, split brain, blindsight, and other paradigms by passionate pioneers in the last century (Seth, 2018), empirical theories of consciousness have emerged in neuroscience. Currently, the situation has reached a critical point of both hope and challenge in that a large number of theories of consciousness (ToCs), each with specific empirical support, have claimed their respective plausibilities, and their proposed conjectures have led to diverging predictions (Del Pin et al., 2021; Signorelli et al., 2021; Seth and Bayne, 2022; Yaron et al., 2022). Various theories have been discussed, and it appears that this issue is becoming more prevalent. Currently, the lack of collaboration between different groups and fields hinders the advancement of theories of consciousness. However, a fundamental theory which is not limited by the boundaries of individual theories is expected to emerge in the future (Koch, 2018).

In this process, four major kinds of ToCs have garnered the most attention (Seth and Bayne, 2022): Integrated Information Theory (IIT) (Tononi, 2008; Oizumi et al., 2014; Tononi et al., 2016), Global Neural Workspace Theory (GNWT) (Dehaene, 2014; Mashour et al., 2020), Higher-Order Theory (HOT) (Lau and Rosenthal, 2011; Brown et al., 2019), and Recurrent Processing Theory (RPT) (Lamme, 2018) and Predictive Processing Theory (PP) (Seth and Hohwy, 2021).

Briefly, IIT identifies any conscious experience with the maximally irreducible cause-effect structure of the system in the corresponding state; GNWT proposes that the global workspace, triggered by widespread neural ignition and the sharing of information across several cognitive modules, is the key to conscious access; HOT is based on the higher-order structure of conscious experience in which “I” am aware of “something” (the representation of “something” is first-order). At the same time, RPT and PP emphasize the importance of top-down processing in conscious mental activity.

Rather than attributing consciousness to neural activities, a fifth approach has identified consciousness with underlying physical processes across multiple spatiotemporal scales. As a typical and noted paradigm, Orchestrated Objective Reduction (Orch OR, cf. Hameroff and Penrose, 2014) theory claims that mental aspects like understanding, free will, or insight cannot be Turing machine computable based on Gödel's incompleteness theorems (Penrose, 1999). It associates consciousness with quantum mechanical processes. The Field Theories of Consciousness, which compare uncertain particle-like and wave-like phenomena as the “neuron–wave duality” (John, 2001), propose that the widespread electromagnetic (EM) fields in brains could be the physical correlates of consciousness (Hunt and Jones, 2023).

Their rivalries are likely to yield a winner through empirical tests (or remove inappropriate theories from the competitive stage to the extent possible) and eventually enable contemporary theories to move toward falsifiable unification (Ellia et al., 2021). Since the preparations begun in 2019, there has been an initial *adversarial collaboration* between IIT and GNWT (Reardon, 2019; Melloni et al., 2021), a project aimed at falsifying various ToCs and breaking down the barriers between them. With the implementation of Chalmers winning the “25-year wager” with Koch on unraveling the mechanism of consciousness at the meeting of the Association for the Scientific Study of Consciousness (ASSC) in 2023, the preliminary result of the adversarial collaboration has been published: neither of them matches their tests perfectly (Lenharo, 2023).

Block (1995) advocated an early distinction between P-consciousness, which focuses on the experiential properties of consciousness (qualia), and A-consciousness, which focuses on the cognitive functions of consciousness (e.g., linguistic activities). Regarding these two aspects of consciousness, GNWT and HOT generally refer to the so-called A-consciousness, whereas IIT and RPT might refer to P-consciousness. This seems to explain why IIT would maintain that the maximum integrated information should be generated in the posterior cortex, whereas the prefrontal cortex, which GNWT emphasizes, would not be necessary for IIT (cf. Koch et al., 2016; Boly et al., 2017; Odegaard et al., 2017). As Doerig et al. (2021a,b) discussed in the hard criteria for testing ToCs, some ToCs associate consciousness singularly with their preferred properties and mechanisms, which are likely to be necessary but insufficient. Similarly, Lamme (2018) comparison of her RPT with other ToCs led to the conclusion that “missing ingredients” exist in all of these necessary theories.

## The trend of unifying the theories of consciousness

In the Chinese context, the classic metaphor of “blind men feeling the elephant” is often used to describe how people each grasp only a particular facet of a thing and therefore perceive the same thing differently because of the discrepancies in the facets to which they have been exposed. In a practical investigation, however, following the method the blind men do may not be such a bad start, as it suggests that we have been exposed to at least parts of the fact and that by correlating this knowledge, we will come to a complete understanding.

The recognized trend toward unifying ToCs has become more widely adopted, such as Wiese (2020) advocating a “minimal unifying model” (MUM) that would be compatible with the major theories. In an attempt to integrate multiple ToCs, Safron (2020) combined IIT, GNWT, and PP to construct a comprehensive theory. This was a remarkable effort, and it would be more explanatory if it incorporated more theoretical and experimental evidence, and could further respond to the conflicts between the remaining theories. As for HOT, Brown et al. (2019) argued that realizing a global workspace requires higher-order metacognition. The Attention Schema Theory (AST) (Graziano, 2019a,b), another current theory of consciousness, has also attracted much attention; Graziano et al. (2020) previously attempted to integrate their AST with GNWT, HOT, and other theories into a standard model of consciousness. In their response, Panagiotaropoulos et al. (2020) agreed with Graziano et al., at least on the orthogonal dimensions of the model of consciousness.

Nevertheless, some cruxes must be considered when comparing and contrasting the various theories. First, we must correctly touch the “elephant” and not something else; otherwise, for example, the integration of a model of finger movements (obviously not consciousness) into a model of consciousness would be troublesome; second, we also need to consider whether the methods or strategies used are appropriate. Regarding ToCs, for the first question, we need to cautiously confirm the diverse global states of consciousness (Bayne et al., 2016; McKilliama, 2020). A transformation in the global state of consciousness would result in a marked shift in the structure of the entire experience, as if going from one inner world to a very different one, rather than a simple change in the intensity or content of the experience. As for the second issue, Lau (2022), in his new book, analyzes in detail the ways in which current experimental methods can lead to biased interpretations of results. The rise of “no-report paradigms” (Tsuchiya et al., 2015), even “no-cognition paradigm” (Block, 2019), recently manifested a practical step forward in this regard.

Being careful of both concerns above, we might effectively have a series of necessary elements if to suppose as A, B, and C… for each indicates a model and corresponding mechanism, such as A referring to IIT. Based on the present approaches to unification, the fundamental theory would be an integration of these elements, i.e.,

the fundamental theory = A and B and C and …

Ideally, this result would be a *necessary and sufficient* condition for consciousness, also referred to as “minimally sufficient” (Fink, 2016). Is this always true? Is it possible that by integrating more and more candidate theories, our model could become more and more accurate? It is also important to note that such attempts at unification often overlook the fifth physical approach.

## The architecture of a layered mind for consciousness

**Figure 1:**
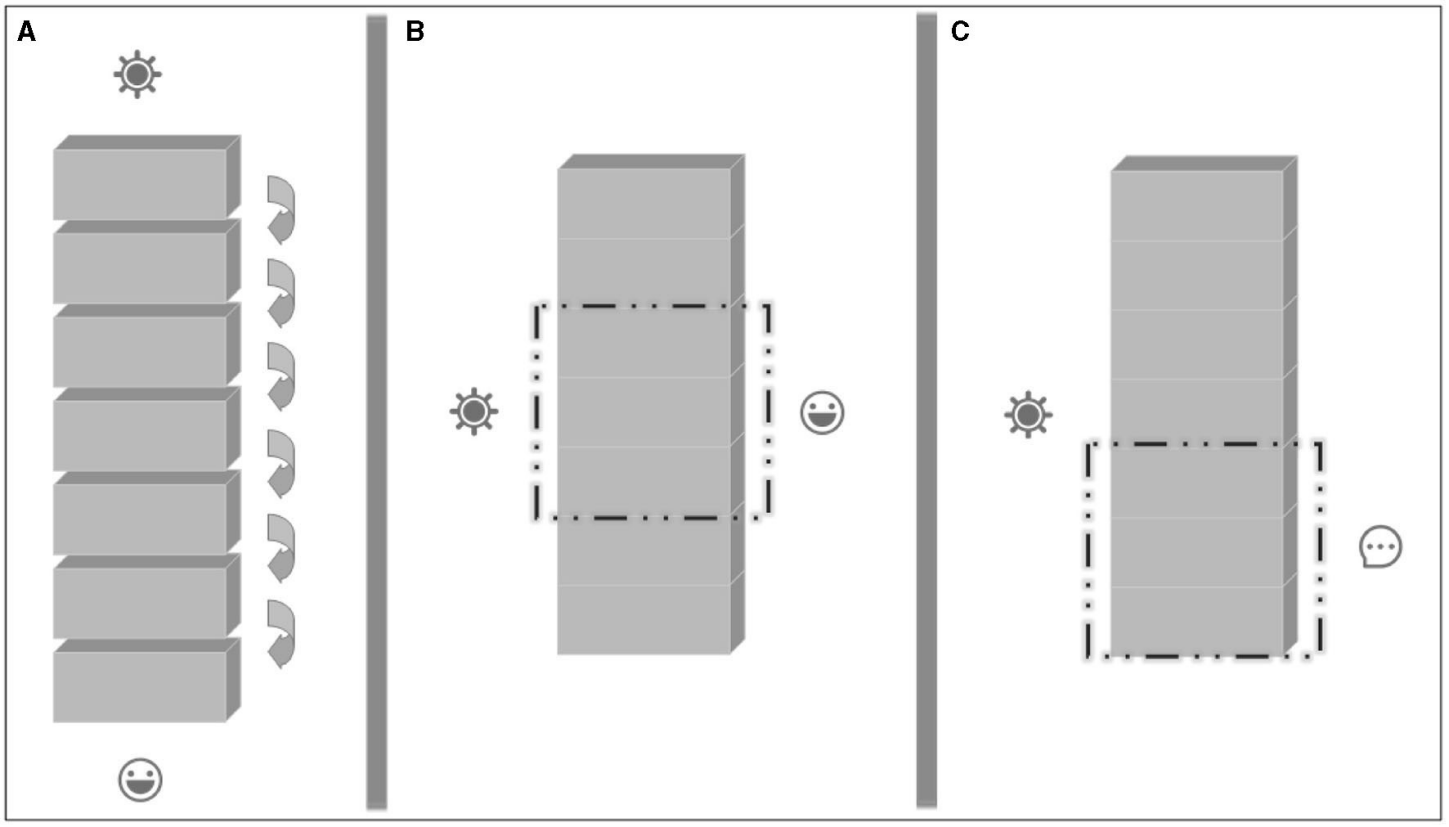
The architecture of layered mind for consciousness. **(A)**: In the architecture of traditional information processing, the stimulus signals are processed sequentially in modules, of which each specific form of information is only the product of a specific processing step, and would finally constitute experience in the so-called imaginary “module of consciousness”; **(B)**: However, in the architecture of layered mind, signals are processed simultaneously in various layers, each of which is a candidate for a temporary “zone of consciousness”; **(C)**: The arbitrary bonds of different layers bring specific types of experiences with different structures and attributes, such as the intervention of higher cognitive functional layers to bring the experience of the conceptual component.

Gazzaniga (2018), “the father of cognitive neuroscience,” suggested his unique view of how consciousness arises based on many instances of abnormal brains he had been exposed to during ward rounds and split-brain research. For machines, confronting a breakdown is a better way for engineers to access and understand how they work. Similarly, neurological diseases indirectly provide an excellent window into the mechanisms of the mind, which Gazzaniga used to explore consciousness in the brain. For his strategy, Gazzaniga considered diverse global states and appropriate methods. He then argued that consciousness is the overall manifestation of the coordination of the diverse basic instincts of the mind, like a symphony without a conductor; in such a distributed system, individuals operate relatively independently, and different combinations of them can exhibit different patterns of performance (see Figure 1).

Unlike other theories, this view does not specify whether cortical activity is sufficient or necessary for consciousness. Gazzaniga found that our brains were resilient. A computer with many severely damaged components would be rendered wholly paralyzed, but the damaged brains in the wards had still been functioning well in a way. There is no palace in the cortex and no part that acts like the core of a computer. Not only are the frontal cognitive modules and the posterior higher sensory cortex candidates for consciousness, but the entire cortex is also an evolutionary expansion of earlier forms of consciousness. In addition to Damasio (2010), Gazzaniga believes that the subcortical affective system may act as an “engine,” with which any cortical module can collaborate to produce a unique conscious experience accompanied by a sense of self. Additionally, Seth (2021) recently endorsed Damasio's illumination of the role of emotion in generating experiences in his theory of consciousness based on PP. If we consider a layered architecture for consciousness, the formulation of the above integration should be

the necessary and sufficient model = the “engine” and (A or B or C or …)

In his recent work, Block (2023) distinguished our perception from cognition, which he used to argue against what he called “cognitive theories of consciousness.” Layered architecture can reconcile this apparent contradiction. From the perspective of the architecture of the layered mind, different global states may result from diverse brain regions and mechanisms. Eventually, both IIT and GNWT, as well as various other important ToCs, will be assessed for their indicative roles within a synthetic model in the meaning of layered architecture.

If we explore this architecture radically into more essential ranges, it may extend to a general version that the physical approach may help out. Our brains, as complex systems, have many components and layers of subsystems, and both Orch OR and EM fields can operate as a hierarchy across multiple levels of the brain. Hameroff (2022) argued the orders of magnitude in frequency in microtubules inside each neuron. The proponents of EM fields describe them from micro to macro scales as “stuff” of phenomenology, patterns of experiences, and phenomenal objects, respectively (Fingelkurts et al., 2013; Hales and Ericson, 2022).

Further work will focus on determining the specific interpretative position of each ToC within the layered model and will help unravel the interaction protocols between the components in the model.

## Discussion

In this opinion article, we reviewed the stalemate that various theories of consciousness, each with its specific empirical support and respective plausibility, and attempted to unify these theories. Contrasting a layered architecture with the unification of traditional viewpoints suggests that it may be a more conducive approach to profoundly understand consciousness and may be compatible with competing theories.

## Author contributions

ZR: Conceptualization, Formal analysis, Methodology, Software, Validation, Visualization, Writing—original draft, Writing—review and editing.
